# Uropathogens antibiotic resistance patterns among type 2 diabetic patients in Kisii Teaching and Referral Hospital, Kenya

**DOI:** 10.11604/pamj.2018.30.286.15380

**Published:** 2018-08-23

**Authors:** Vincent Mogaka Mageto, Mathenge Scholastica Gatwiri, Wachuka Njoroge

**Affiliations:** 1 Department of Medical Laboratory Sciences, Kenyatta University, Nairobi, Kenya

**Keywords:** Type 2 diabetes mellitus, urinary tract infections, antibiotic sensitivity, E, coli, K, pneumoniae

## Abstract

**Introduction:**

Non-insulin dependent diabetes mellitus is a major risk factor for urinary tract infections. Irrational use of antibiotics has led to the emergency of uropathogens resistant to available antibiotics. The main objective was to determine the bacterial causative agents of urinary tract infections and their antibiotic resistance patterns.

**Methods:**

One hundred and eighty (180) type 2 diabetic patients were recruited to take part in the study. Urine samples were collected and cultured for urinary tract infections diagnosis and antibiotic sensitivity.

**Results:**

A total of 35 isolates were obtained from the study. All the isolates were sensitive to gentamicin. All 21 (100%) isolates of *E. coli* were sensitive to gentamicin and cephalexin. All 10 (100%) *K. pneumoniae*isolates were sensitive to gentamicin and nitrofurantoin. Out of the 21 *E. coli* isolates, five of them showed resistance to ampicillin, three *E. coli* isolates showed resistance to nitrofurantoin and another three *E. coli*isolates showed resistance to co-trimoxazole. Out of 10 *K. pneumoniae* isolates, two of them were found to be resistant to ampicillin, one *K. pneumoniae* isolate was resistant to cephalexin and two *K. pneumoniae*isolates were resistant to co-trimoxazole. Out of the four *P. mirabilis* isolates, there were three cases where one isolate was each resistant to ampicillin, nitrofurantoin and co-trimoxazole.

**Conclusion:**

There is a need to have a regular screening of bacterial isolates causing urinary tract infection in diabetic patients and their antibiotic sensitivity in order to have effective therapy. Present findings show that there is increased resistance to the commonly prescribed antibiotics.

## Introduction

Urinary tract infection is the main cause of morbidity among diabetic patients [1]. Diabetes mellitus is associated with bladder dysfunction, glycosuria and low immunity, all of which predispose an individual to UTI [2, 3]. *E. coli* is the leading isolate in UTI cases. The increasing numbers of *K. pneumoniae* isolation in UTI cases pose a heavier burden of managing UTI in diabetic patients. Development of antibiotic resistance is a big threat to diabetic patients who are already predisposed to UTI and who in most times have dysfunctional urinary tracts prompting for instrumentation. Complications associated with diabetes are increasingly becoming of interest due to their alarming mortality rate as ranked by the World Health Organization [4]. Understanding the resistance patterns of bacterial isolates will be of great help to health professionals in the management of UTI. Uncontrolled antibiotic use is posing a great threat to the treatment of bacterial infections. Some of the available antibiotics that are administered are not able to kill bacterial pathogens. Most nations in Africa are greatly affected by the emergence of multidrug-resistant strains because of the inadequate surveillance and prompt management [5]. Studies in Africa have shown the need to have a systematic screening of UTI in diabetic patients due to the increasing prevalence. In Sudan, symptomatic and asymptomatic bacteriuria among diabetic patients was found to be 17.1% and 20.9% respectively [6]. There is limited data on the antibiotic susceptibility of bacterial isolates causing UTI among diabetes patients in Kenya. This study will provide some of the missing data and probable solutions. This study was carried out with the aim of isolating bacterial pathogens responsible for UTI among diabetic patients and their antibiotic sensitivity patterns.

## Methods

The study was conducted in Kisii teaching and Referral Hospital in Kenya. The referral facility serves patients from south Nyanza, rift valley and western counties in the country. All participants in this study were known type 2 diabetic patients visiting the health facility. The inclusion criteria was based on type 2 diabetic patients who had not been diagnosed with UTI and were not on antibiotic therapy for the last two weeks. Exclusion criteria was individuals already diagnosed with UTI and those on antibiotic therapy. Being on antibiotic therapy during the period of study would have a suppressing effect on growth of pathogens and hence affecting their sensitivity towards available antibiotic agents. Type 1 diabetes mellitus constitutes less than 4 percent of the total diabetes cases and therefore type 1 diabetes mellitus patients were also excluded. The study was carried out between August and December 2017. A cross-sectional study was adopted. Bacteria pathogens were isolated, identified and tested for antibiotic sensitivity using the standard procedure [**7**]. Clean catch midstream morning urine samples were collected from all participants. The specimens were cultured on Cysteine Lactose Electrolyte Deficient Agar (Rapid labs) and incubated at 37^o^C for 18-24 hours. American Type Culture Collection *S. aureus*25923 and *E. coli* ATCC 25922 were used as controls for the prepared culture plates. Gram stain was done on significant colonies followed by biochemical tests to identify the species of the bacteria. *E. coli* was identified as a gram-negative rod, lactose fermenter, indole positive and negative citrate test while *K. pneumoniae* was identified as a gram-negative rod, lactose fermenter and positive citrate test. Proteus mirabilis was identified as gram-negative rods, non-lactose fermenter and indole negative. Antibiotic sensitivity testing was carried out using the Kirby Bauer technique in accordance with the EUCAST standards [**8**]. Main drugs implicated in the sensitivity testing were penicillin, aminoglycosides and cephalosporins. The actual drugs used were ampicillin (10 ug), gentamicin (10 ug), nitrofurantoin (100 ug), cephalexin (30 ug) and co-trimoxazole (25 ug).

**Ethics clearance**: This study was carried out in accordance to the declaration of Helsinki and International Conference on Harmonization Guideline on Good Clinical Laboratory Practice (ICH-GCLP). The protocol and informed consent form were reviewed and approved by the Kenyatta University Ethics Review Committee (PKU/610/I694 on 18^th^ May 2017). The research permit was granted by National Commission for Science, Technology and Innovation (NACOSTI/P/17/88572/17706 ON 6^th^ July 2017) Kenya. Written informed consent was then obtained from each participant. A unique identification code was assigned to every participant to ensure confidentiality of the findings. All records were stored in a locked cabinet in a secure room accessible to the principal investigator.

## Results

Out of the 180 participants, 37 tested positive for urinary tract infection. The prevalence of UTI was, therefore, found to be 20.6%. As shown in Table 1, antibiotic sensitivity revealed *P. mirabilis* to be the most resistant gram-negative isolate to ampicillin (25%), nitrofurantoin (25%) and cotrimoxazole (25%). All isolates were sensitive to gentamicin (100%). Only one *K. pneumoniae* isolate showed resistant to cephalexin (10%) of all gram-negative isolates. The findings in this study showed a similar trend in others studies concerning resistance patterns [6, 9]. Table 2 has findings from this study showing multi-drug resistance to be a threat to the management of UTI in diabetes mellitus. When considering antibiotic resistance against two antibiotic agents used in the study, multi-drug resistance was found to be at 13.5%. Three *E. coli* isolates and two *K. pneumoniae* isolates were found to be resistant to two antibiotics. When considering resistance against three of the four antibiotic agents used, multi-drug resistance was also found to be 13.5%. However, in this case, we had three *E. coli* isolates, one *K. pneumoniae* and *P. mirabilis* isolate each. None of the isolates showed resistance to four or five of the antibiotics used in the study.

**Table 1:** Antibiotic resistance patterns of bacterial isolates

Isolate (n)	Antibiotic agent				
	AP (%)	GM (%)	NF (%)	CEX (%)	CTZ (%)
*E. coli* (21)	23.8	-	14.3	-	14.3
*K. pneumoniae* (10)	20	-	-	10	20
*P. mirabilis* (4)	25	-	25	-	25

Key: AP = ampicillin; GM = gentamicin; NF = nitrofurantoin; CEX = cephalexin; CTZ = cotrimoxazole

**Table 2:** Bacteria resistance to two and three antibiotic agents

	Resistance to antibiotic agents	
No. of organism	R2	R3
*E. coli* (n=21)	14.3%	14.3%
*K. pneumoniae* (n=10)	20%	10%
*P. mirabilis* (n=4)	0%	25%

**R2** = Resistance to two antibiotic agents

**R3 =** Resistance to three antibiotic agents

## Discussion

This study showed that bacterial pathogens causing UTI among diabetics exhibit high multi-drug resistance rates. This has also been revealed in different studies conducted in various regions like Sudan, United Arab Emirates, Ethiopia, Libya, Australia and Israel [6, 9-13]. A study carried out in Ethiopia showed that over 60% of the isolated *E. coli* was resistant to ampicillin [10]. Most multi-drug resistant cases of *E. coli* had been reported among pregnant women in Ethiopia [10]. While findings of this study showed a multi-drug resistance of up to three antibiotic agents used, a similar study in Sudan revealed multi-drug resistance of up to four antibiotic agents used [6]. A study carried out in Australia concluded that routine use of antibiotics in primary care could be a cause of antibiotic resistance [14]. There are some factors that contribute towards the development of antibiotic resistance in different geographical locations. The frequency of antibiotic use and adherence to prescribed doses are some of the factors that can vary from place to place. This study showed *K. pneumoniae* resistance against ampicillin and cotrimoxazole to be at 20%, a rate which is comparable to the findings in a study done in Sudan 22.2% [6]. The resistance of *P. mirabilis* against ampicillin, nitrofurantoin and cotrimoxazole was found to be at 25% while a similar study in Sudan found it to be at 33% [6].

In a retrospective study done in South Africa between 2004 and 2009, *P. mirabilis* showed the highest resistance to ampicillin, cloxacillin, amoxicillin, tetracycline, co-trimoxazole, erythromycin and chloramphenicol [15]. *Proteus mirabilis* is a common pathogen in nosocomial infections and its resistance has been on the rise in the recent past. The frequency of antibiotic use in most countries has been found to be the reason for the increasing antibiotic resistance. Studies have also found out that missing information in the resistance patterns in many developing nations contributes to the burden of management of antibiotic resistance. A study carried out in Indonesia concluded that there is an urgent need to have up to date findings on current antibiotic resistance patterns as this helps in interventions aimed at management of antibiotic resistance [16]. The economic burden that comes along with antibiotic resistance was also explained. Most of the affordable antibiotics that are readily available to individuals are not effective anymore due to resistance [16]. It should be noted that even if 100% gentamicin sensitivity was recorded, caution must be taken before they are used. Aminoglycosides should not be used in patients with chronic kidney diseases due to their nephrotoxic effect. Just like other aminoglycosides, gentamicin decreases the glomerular capillary filtration by destroying the proximal tubule cells.

## Conclusion

In conclusion, our study points out the need to have frequent and extensive screening for urinary tract infections and antibiotic resistance among diabetic patients. There is limited data on antibiotic resistance among diabetic patients in Kenya and this is becoming a stumbling block to the proper and efficient management of urinary tract infections and the fight against antibiotic resistance in general. The study recommends that more studies should be carried out in other regions in Kenya and more classes of antibiotics should be used in future studies so that diverse antibiograms can be developed and help in the treatment of urinary tract infections among diabetic patients. Additionally, dose adjustment is critical as some antibiotics may be toxic to the kidney and hence worsening the condition of patients with chronic kidney diseases. Gentamicin, for instance, recorded 100% sensitivity in this study but it is nephrotoxic and should be used with caution.

### What is known about this topic

- *E. coli* is the leading isolate in urinary tract infections;
- Diabetic patients are prone to urinary tract infections.

### What this study adds

- In the study, among the three bacterial agents identified, each had an isolate that was resistant to three antibiotic agents used suggesting the increasing antibiotic resistance;
- The high resistance pattern shown by *P. mirabilis* suggests that even bacterial pathogens that are not common causative agents of UTI among type 2 diabetic patients are turning out to be highly resistant to available antibiotics.
